# Enhancing antimicrobial resistance surveillance and research: a systematic scoping review on the possibilities, yield and methods of data linkage studies

**DOI:** 10.1186/s13756-025-01540-7

**Published:** 2025-03-29

**Authors:** S. A. M. van Kessel, C. C. H. Wielders, A. F. Schoffelen, A. Verbon

**Affiliations:** 1 Centre for Infectious Disease Control (CIb), National Institute for Public Health and the Environment (RIVM), Bilthoven, The Netherlands; 2 Department of Internal Medicine, University Medical Centre Utrecht, Utrecht, The Netherlands

**Keywords:** Antimicrobial resistance, Epidemiology, Surveillance data, Data linkage, Scoping review

## Abstract

**Background:**

Surveillance data on antimicrobial resistance (AMR) determinants such as antibiotic use, prevalence of AMR, antimicrobial stewardship, and infection prevention and control are mostly analysed and reported separately, although they are inextricably linked to each other. The impact of surveillance and research can be enhanced by linking these data. This systematic scoping review aims to summarize the studies that link AMR data and evaluate whether they yield new results, implications, or recommendations for practice.

**Methods:**

For this review, data linkage is defined as the process of linking records, from at least two independent data sources on either (I) at least two AMR determinants or (II) one AMR determinant and one or more general population characteristics. Data linkage should be performed on the level of a certain entity which, in the context of this review, can encompass persons, healthcare institutes, geographical regions or countries. A systematic literature search was performed on February 7th 2025 in Embase.com, PubMed and Scopus to identify AMR data linkage studies.

**Results:**

Forty-eight articles were included in our review. Most data linkage studies used two data sources, and most studies were published in the last 5 years (n = 23 in 2020–2024). A predominance of studies linked data on geographical location, and thirteen studies linked data on individual patient level. Findings demonstrate that the majority of studies (43/48) had added value and provided recommendations for clinical practice and future policies or had suggestions for further research and surveillance. Additionally, data linkage studies appeared to be suitable for hypothesis generating. Several limitations were identified. Most studies had ecological designs, which are prone to ecological fallacy and unobserved confounding, making it hard to establish causality.

**Conclusion:**

This systematic scoping review showed that AMR data linkage studies are increasingly performed. They have potential to gain a more comprehensive understanding of AMR dynamics by generating hypotheses, assisting in optimisation of surveillance, and interpretation of data in the context of guideline/policy development. To increase the added value of data linkage, more studies should be performed to improve knowledge on methodological approaches, data access, data management, and governance issues.

**Clinical trial number:**

Not applicable.

**Supplementary Information:**

The online version contains supplementary material available at 10.1186/s13756-025-01540-7.

## Background

Antimicrobial resistance (AMR), has emerged as one of the leading public health threats worldwide [1]. Appropriate antibiotic use and the prevention of transmission of resistant bacteria are the cornerstones in the control of AMR. Reliable epidemiological information about the prevalence and impact of AMR is essential to implement practical and focused measures regarding antibiotic use and infection prevention. Although they are inextricably linked to each other, data on AMR determinants such as antibiotic use, prevalence of AMR, antimicrobial stewardship (AMS), and infection prevention and control (IPC) are mostly analysed and reported separately. The impact of surveillance and research can be enhanced by combining data on these different AMR determinants, as well as by combining these data with data from population characteristics [2]. Linking data sources on for example institutional level or geographical location gives the opportunity to identify correlations and trends between different determinants. These insights could help in defining new hypotheses and contribute to rational adaptation of e.g. clinical guidelines or local/national IPC practices. However, it is not clear to what extent AMR data linkage studies have been performed, what their challenges are, and what their yield is.

Several cases illustrate the advantage of linking AMR data from different data sources. As an example, the European Union agencies deliver joint inter-agency antimicrobial consumption and resistance analysis (JIACRA) reports [3]. They analyse data from humans and food-producing animals on AMR and antibiotic use, which provides valuable insights for policymakers. Another example is the study of Lishman et al. [4], which linked prescription data of first-line antibiotics to incidence data of resistant urinary tract infection (UTI) related bacteraemia, on the level of primary care practice. Indeed, the antibiotics that were prescribed more frequently were associated with higher incidences of resistant bacteria causing bloodstream infections. The results indicate that a reduction in the prescriptions for UTIs in primary care could lead to a decrease in resistant bacteria causing infections. Additionally, a data linkage study assessing the effect of antibiotic use on AMR at a country level [5] found an immediate increase and persistent upward trend in AMR, following a rise in antibiotic use in the same or a neighbouring country, highlighting the need for international cooperation and policies to discourage overuse of antibiotics. These examples underscore the potential of linking AMR data.

Here, we conducted a systematic scoping review to investigate the extent to which AMR data linkage studies have been performed and to identify their challenges. Our focus is on the yield and added value of merging different AMR-related data sources, including (1) recommendations for clinical practice, (2) implications for guiding future policies, (3) suggestions for future research, and (4) suggestions for surveillance.

## Methods

This systematic scoping review was conducted following the methods outlined by Arksey & O’Malley [6]. Results were reported according to the Preferred Reporting Items for Systematic Reviews and Meta-Analysis Extension for Scoping Reviews (PRISMA-ScR) Statement, for which a checklist is provided in Additional file 1. [7].

### Definition of data linkage

For the purpose of this review, data linkage is defined as the process of linking records, from at least two independent data sources on either (I) two or more AMR determinants or (II) at least one AMR determinant and one or more general population characteristics. Data linkage should be performed on the level of a certain entity which, in the context of this review, can encompass persons, healthcare institutes, geographical regions or countries. Years and other defined time periods are not considered appropriate entities for data linkage.

### Search strategy

The search strategy was developed in consultation with an experienced information specialist. We focused on three main concepts: (1) terms related to data linkage, (2) terms related to AMR and antibiotic use, and (3) terms related to AMS. A combination of synonyms and wildcards was used to ensure a comprehensive search. Records needed to refer to concept 1 and either concept 2 or concept 3 in the title to be included. The final search strategy can be found in Additional file 2, with the different concepts given in different colours. Scientific databases Embase.com, PubMed and Scopus were searched for relevant articles on February 7th, 2025. There were no publication date restrictions. The identified records were exported to the citation management program Endnote (version 21.0.1), and duplicate records were removed.

### Inclusion and exclusion criteria

Studies were eligible for inclusion if the method of data linkage met our definition as described above. We focused only on studies on antibiotic resistance, i.e. studies on antivirals, antifungals, and antiparasitics were excluded. In addition, studies were considered eligible if their study aim focused on determining the effect of AMS interventions, antibiotic use, other AMR determinants, or general population characteristics on an AMR related outcome (for instance AMR prevalence or antibiotic use). At least one of the AMR determinants included in the studies should concern human data. Studies for which additional data collection as a new data source was performed, e.g. by sending out questionnaires, could be included, but at least one data source should already have been existing in advance.

Studies were excluded if the data source used for linkage was a literature review. Furthermore, studies were excluded when no full text was available in English, and when the design concerned a clinical trial, meta-analysis, or systematic review.

### Study selection

Titles and abstracts of records resulting from the systematic literature search, were screened for eligibility independently by two reviewers (SK and CW). Online software Rayyan was used. Discrepancies were solved by consensus with the help of a third reviewer (AS). Subsequently, the full texts of the reports were read (SK read all papers, CW and AS each read 50% (to a total of 100%)) and a final selection for inclusion was made.

### Data charting

A data charting form was developed to guide the data charting process. The extracted variables comprised general study characteristics like title, first author, year of publication, country, and research question. In addition, the number and types of data sources used, level of data linkage, and the type of analysis performed were extracted from the studies. Lastly the key findings, recommendations and implications for use, and the strengths and limitations regarding data linkage were recorded. One reviewer (SK) charted the data and the results were verified by two other members of the team (CW and AS) by cross-checking selected sections of the data against the original articles to ensure accuracy. Extracted data were interpreted.

## Results

### Study selection

**Fig. 1:**
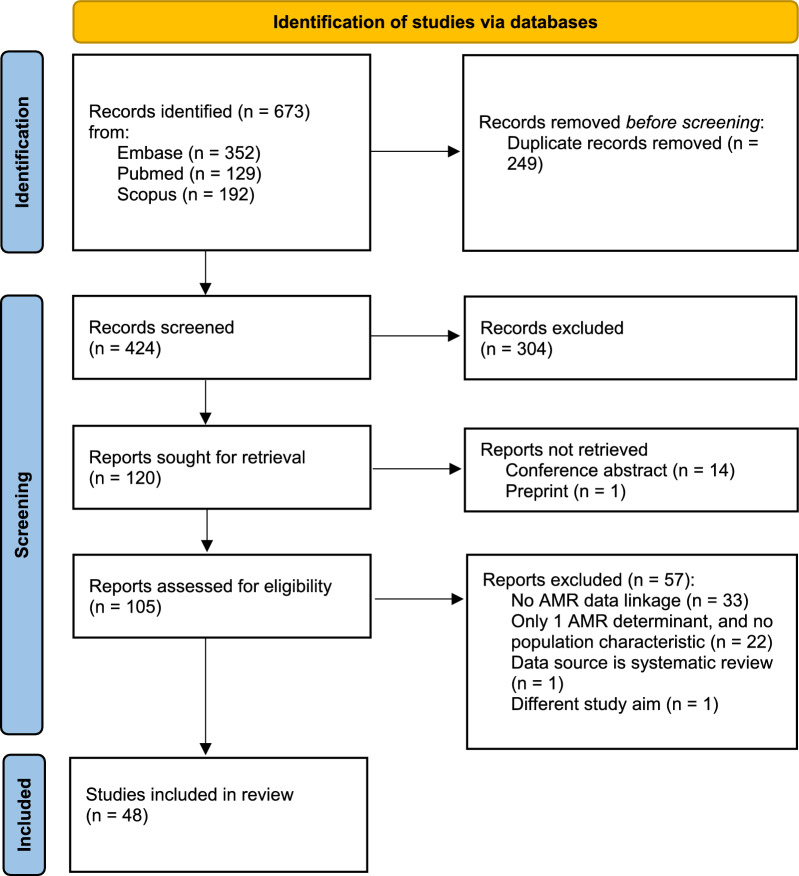
Flow diagram of study selection (*From:* Page et al. [8]

The literature search yielded 673 records, of which 249 duplicates were removed. After screening title and abstract, another 304 records were excluded. Fifteen records could not be retrieved. One hundred and five reports were assessed for eligibility. Fifty-seven reports were excluded because they did not match the eligibility criteria. The most common reason for exclusion was that the study diverged from our definition of a data linkage study (n = 33). For instance, some studies linked data from a single source, such as different parts of electronic patient files, or linked data at a year level. Other reasons for exclusion were the use of only one AMR determinant and no data on general population characteristics (n = 22), the use of data from a systematic review (n = 1), and a different study aim being creating a web-based application (n = 1). In total, 48 articles were included in this scoping review. A summary of the article selection process is provided in a PRISMA flowchart (Fig. 1).

### Study characteristics

Of the 48 identified AMR data linkage studies, the majority had an ecological design, characterized by group-level data and aggregated measures, often used to explore potential associations. Most articles were published in the last five years (n = 23 in 2020–2024). The oldest data linkage study found was published in 1998. Fifteen studies linked data from multiple countries, of which nine included only European countries and six included data from countries from multiple continents. Most single-country studies were performed in England (n = 8) and the United States (n = 8), followed by the Netherlands (n = 3) and Japan (n = 3).

More than half of the included studies linked data on geographical locations. Specifically, thirteen studies used country-level data, and fourteen studies linked data on the regional level, which varied from province or district to city level. The remainder of the studies linked data on the level of individual patient (n = 13), primary care practice (n = 4), hospital (n = 1) or long-term care facility (n = 1). Two studies used two different levels of data linkage in their methods, namely patient level and either primary care level or long-term care facility level [9].

We identified different categories of AMR data linkage studies based on the data sources used for linkage. An overview of study characteristics and study aims, stratified by the identified categories, is presented in Table 1. Studies that linked data on antibiotic use and AMR (n = 9) mainly answered research questions regarding the association between the consumption of certain antibiotics and the prevalence of resistance to these antibiotics. Studies that linked data on population characteristics with data on antibiotic use and/or AMR (n = 30) aimed to evaluate the association between demographic, economic or governance factors and antibiotic use and AMR. Examples of these factors are gender, age, socio-economic status, education level, ethnicity, knowledge on antibiotic use, universal influenza immunization, access to drinking water, travel history, ambient temperature and cultural differences.

**Table 1:** Overview of characteristics of data linkage studies, stratified by categories based on data sources used

Author	Year	Location	Level of data linking	Number of data sources used	Study aim
*Antibiotic use* + *AMR (n* = *9)*					
Clifton et al. [10]	2018	England	Patient	3	Association between azithromycin exposure and *Neisseria gonorrhoea* azithromycin susceptibility
Hirabayashi et al. [11]	2020	Japan	Region	2	Association between the frequency of *Escherichia coli* and *Klebsiella pneumoniae* isolates with an IMP-6 phenotype and usage of carbapenems, fluoroquinolones, and third-generation cephalosporins
Houben et al. [12]	2014	The Netherlands	Hospital	2	AMR and use of selective digestive decontamination/selective oropharyngeal decontamination in intensive care units
Kenyon et al. [13]	2020	Europe	Country	2	Association between antibiotic consumption and AMR in *Neisseria gonorrhoeae*
Kenyon et al. [14]	2020	Europe	Country	2	Association between prevalence of NG-MAST genogroups associated with decreased susceptibility to cephalosporins and fluoroquinolones and consumption of these antibiotics
Manoharan-Basil et al. [15]	2022	Europe	Country	2	Association between consumption of quinolones and cephalosporins and the time-lagged prevalence of resistance to these antimicrobial classes
McDonnell et al. [16]	2024	England	Region	2	Association between antibiotic prescribing intensity and rates of UTI resistance
Pouwels et al. [17]	2019	England	Region	2	Evaluation of selection and co-selection by antibiotic use among *Escherichia coli* isolated from urinary samples
Rahman et al. [5]	2023	Europe	Country	3	Long-term effect of antibiotic use on AMR
*Antibiotic use* + *AMR* + *population characteristics (n* = *7)*					
Boszczowski et al. [18]	2020	Brazil	Region	4	Impact of overall antibiotic use on the incidence of bloodstream infections in intensive care units, adjusted by socioeconomic factors, and quality and access to healthcare
Buczkowska et al. [19]	2024	England	Patient	2	Linking data from patient questionnaires and genome sequencing (including AMR profiles) from enteric fever cases
Maugeri et al. [20]	2023	Europe	Country	3	Evaluating how demographic, economic, governance, health and freedom characteristics contribute to antibiotic consumption and AMR
Maugeri et al. [21]	2023	Europe	Country	4	Association between temperature change and AMR, considering antibiotic use, population density, gross domestic product per capita and governance indicators
Terahara et al. [22]	2019	Japan	Region	3	Assessing the correlation between fluoroquinolone consumption and levofloxacin resistance in *Escherichia coli*
Terahara et al. [23]	2019	Japan	Region	3	Assessing associations between carbapenem use and the prevalence of imipenem/meropenem resistance in *Pseudomonas aeruginosa*
Van Bijnen et al. [24]	2015	Europe	Primary care practice	3	Assessing risk factors for nasal carriage of resistant *Staphylococcus aureus* including ecological exposure to antibiotics
*Antibitoic use* + *population characteristics (n* = *10)*					
Kenyon et al. [25]	2020	Worldwide	Country	3	Association between antibiotic consumption, governance and cultural traits
Kim et al. [26]	2023	US	Patient	2	Association between antibiotic prescription and self-reported sociodemographic characteristics
Kwong et al. [27]	2009	Canada	Region	3	Evaluating the effect of universal influenza immunization on antibiotic prescriptions
Norris et al. [28]	2005	New Zealand	Region	2	Association between antibiotic use and gender, age, and socioeconomic status
Opatola et al. [29]	2024	Wales	Patient	5	Association between child weight and repeated antibiotic prescription
Patterson et al. [30]	2019	Ireland	Patient	2	Compare antibiotic prescribing in different settings (care home vs community) to inform AMS interventions
Sarpong et al. [31]	2015	US	Region	2	Relationship between individual, family, and community level characteristics on antibiotic use
Schuts et al. [32]	2019	The Netherlands	Patient	2	Associations between appropriate knowledge on antibiotics, ethnicity, and antibiotic use
Tarkhashvili et al. [33]	2023	US	Region	2	Effect of the prevalence of poverty on antibiotic prescribing rates
Vo et al. [34]	2025	US	Patient, Long term care facility	4	Association between facility-level antibiotic prescribing, individual-level factors and antibiotic use among nursing home residents
*AMR* + *population characteristics (n* = *13)*					
Aliabadi et al. [9]	2022	England	Patient, Primary care practice	4	Creating a comprehensive epidemiological picture of *Escherichia coli* bacteraemia trends and risk factors
Francois Watkins et al. [35]	2024	US	Patient	2	Influence of patient and isolate factors on clinical outcomes of infections with resistant or susceptible *Salmonella*
Chen et al. [36]	1998	US	Region	2	Association between socioeconomic status and increased risk of drug-resistant invasive pneumococcal infections
Cohen et al. [37]	2014	Worldwide	Country	5	Factors associated with transmission of highly drug resistant tuberculosis
Cordova et al. [38]	2004	Australia	Patient	2	Factors associated with methicillin-resistant *Staphylococcus aureus* infection
Fuhrmeister et al. [39]	2023	Worldwide	Region	4	Relationship between AMR and community access to drinking water and sanitation
Grass et al. [40]	2019	US	Patient	2	Association between quinolone susceptibility and international travel
Li et al. [41]	2023	China	Region	2	Association between ambient temperature and AMR
Ljung et al. [42]	2011	Sweden	Patient	2	Geographical and educational differences in fluoroquinolone prescription in the treatment of urinary tract infection
Mollendorf et al. [43]	2014	South Africa	Patient	3	Risk factors for pneumococcal ceftriaxone nonsusceptibility
Shiferaw et al. [44]	2012	US	Patient	2	Identification of predictors of AMR in *Shigella* isolates
Singh et al. [45]	2009	Canada	Patient	2	Demographic and risk behaviour information and AMR in *Neisseria gonorrhoeae*
Zhen et al. [46]	2021	China	Region	2	Association between AMR and socioeconomic factors
*Antibiotic use* + *AMR* + *animal data (n* = *5)*					
Allel et al. [47]	2023	Worldwide	Country	Not clear, at least 10	Associations between socioeconomic, anthropogenic, and environmental indicators of AMR in humans and food-producing animals
Kenyon et al. [48]	2021	Worldwide	Country	3	Association between fluoroquinolone use in animals and fluoroquinolone resistance in human pathogens
Kenyon et al. [49]	2022	Worldwide	Country	3	Association between use of macrolides in food-producing animals and the prevalence of macrolide resistance in *Streptococcus pneumoniae* in humans
Vieira et al. [50]	2011	Europe	Country	3	Correlations between AMR in *Escherichia coli* blood stream infections in humans and animals, and antibiotic use
Zhang et al. [51]	2019	Europe	Country	5	Factors related to fluoroquinolone resistance and antimicrobial consumption in humans and animals
*Antibiotic use* + *AMR* + *third (n* = *4)*					
Aliabadi et al. [52]	2021	England	Primary care practice	4	Effectiveness of a national antimicrobial stewardship intervention on *Escherichia coli* bacteraemia
Ironmonger et al. [53]	2018	England	Primary care practice	4	Effect of general practice characteristics and antibiotic prescriptions on AMR among *Escherichia coli* from urine
Lishman et al. [4]	2018	England	Primary care practice	3	Association between use of trimethoprim and nitrofurantoin and the incidence of (resistant) urinary tract infection related bacteraemia
Verhoef et al. [54]	2016	The Netherlands	Long term care facility	3	Predictors of AMR in long-term care facilities

*AMR* Antimicrobial Resistance, *AMS* Antimicrobial Stewardship, *NG-MAST* Neisseria gonorrhoeae multi-antigen sequence typing

### Data sources

Most included data linkage studies used two (n = 23) or three (n = 14) data sources. Other studies linked data from four (n = 7), five (n = 3), or more sources (n = 1). For the determinant AMR, antimicrobial susceptibility testing (AST) data was used most frequently as a database (n = 25). Another eleven studies that used AMR data, did not explicitly mention whether the database included individual test values or only aggregated numbers per entity. For the determinant antibiotic use, prescription data was the most common used data type (n = 13), but also antimicrobial sales data was used in several studies (n = 8). Additionally, insurance data and patient charts were used. Ten studies stated that they used antimicrobial consumption data. It is not clear how ‘consumption’ was measured, since this could not be extracted from the papers. Data on population characteristics was mostly obtained through demographic databases (n = 23) and population surveys (n = 9). In addition to existing data, some studies performed extra questionnaires. Other sources that were used contained data on institution characteristics or primary care practices.

### Added value

Forty-three studies (90%) identified added values, aligning with the categories specified in our aim. Thirteen articles even described added values related to two categories. An overview of added values, stratified by different categories of AMR data linkage studies, is given in Table 2. It is noticed that there is quite some overlap in recommendations for clinical practice and implications for future policies. A distinction was made based on whether the recommendation was addressed to caregivers or policy makers.

**Table 2:** Practical deliveries identified in AMR data linkage studies

	Recommendations for clinical practice	Implications for guiding future policies	Suggestions for further research	Suggestions for surveillance
Antibiotic use + AMR (n = 9)	Take into account certain patient characteristics and local conditions in AMS programs and/or antibiotic prescribing [13–15, 17] Reduce (overall) antibiotic prescription/consumption [14, 16, 17] Use different antibiotic as first-line treatment [17]	More international cooperation [5] Need for policy to discourage overuse of antibiotics [5] Support development of new antibiotics [5]	Further research on whether found associations hold in other settings [10, 12] Update the study with more data [11] Examine the role of patient related factors [15] Perform individual-level studies [16]	
Antibiotic use + AMR + population characteristic (n = 7)		Preferred level of action is national [24] Increase governance efficiency at global level [20]	Study from one health perspective [20] Other study design to confirm causal relationship [22] Examine the role of patient related factors [18] Update the study with more data [19]	Use of data linking show potential to monitor causal links in longitudinal manner [23]
Antibiotic use + population characteristic (n = 10)	Take into account certain patient characteristics and local conditions in AMS programs and/or antibiotic prescribing [29–31, 33, 34]	Take into account local context in AMS programs [25, 32] Improve awareness in care home setting [30] Consider influenza vaccination to decrease antibiotic use [27]	Update the study with more data [32] Replicate findings over time [34]	
AMR + population characteristic (n = 13)	Take into account certain patient characteristics and local conditions in AMS programs, AST, and antibiotic prescribing [40, 45, 46]	Take population characteristics and/or local conditions into account for policy attentions [39, 41] Ongoing efforts to limit entry and spread of resistant strains in environmental and healthcare settings [35] Implement quality indicators on antibiotic prescription on national or regional levels [42]	Examine the role of patient related factors [36] Examine the role of strain factors on clinical outcomes [35] Other study design to confirm causal relationship [39]	Surveillance of resistance is recommended to ensure empirical treatment guidelines are appropriate [43, 45] Increase investment in surveillance and improve treatment capacity [37] Make surveillance systems flexible to simplify implementing new elements [45] Surveillance for specific patient group [40]
Antibiotic use + AMR + animal data (n = 5)	Reduce antibiotic use in animals [47, 48]	Integrated approach: focus on social development and poverty reduction as well [47] Closer medico-veterinary collaboration to create guidelines to promote reducing antibiotic use [51]	Update the study with more data [51] Perform individual-level studies [48, 49]	Surveillance of resistance among animal [50]
Anticiotic use + AMR + third (n = 4)	Take into account certain patient characteristics and local conditions in antibiotic prescribing [53] Use different antibiotic as first-line treatment [4] Reduce antibiotic prescription [4, 53]		Perform individual-level studies [4]	Surveillance of resistance genes is recommended [52] Integrated surveillance (linking data on antibiotic use, microbiological testing, clinical background data and epidemiological data) [54]

*AMR* Antimicrobial resistance, *AMS* Antimicrobial stewardship, *AST* Antimicrobial susceptibility testing

In total, sixteen of the identified data linkage studies gave recommendations for clinical practice, such as taking into account certain patient characteristics and local conditions in AMS programs and antibiotic prescribing. Furthermore, a common recommendation was prudent antibiotic prescribing and the use of a different antibiotic as first-line treatment. Studies including population characteristics also often led to implications for guiding future policies, which were found in thirteen articles and many studies recommended to take population characteristics into account. Examples are increasing policy attention for specific regions, changing prescribing guidelines for certain groups, tailoring AMS campaigns to local context, and improving awareness of AMR in specific settings. Eighteen studies provided suggestions for further research in their discussion. The most common suggestion for further research was to update the study with more data, and data on patient characteristics that were not yet included or were only used as covariates in the model. Other suggestions were that other designs and individual-level studies should be used to confirm a causal relationship. Suggestions for surveillance (eight studies) were reported mostly in studies on AMR and population characteristics. Most studies emphasized the importance of surveillance data as being essential for developing risk management strategies, appropriate empirical treatment guidelines, regular analysis, evaluation of interventions and for early response of emerging trends.

### Strengths and limitations of data linkage studies

Several strengths and limitations with regard to data linkage were identified in the studies. In twelve studies a strength of data linkage was explicitly mentioned and in 30 studies at least one barrier was explicitly mentioned. Strengths and limitations are summarized in Table 3.

**Table 3:** Identified strengths and limitations of data linkage

Strengths	Limitations
Useful for hypothesis generating [36–38, 48]	Ecological fallacy [4, 13, 14, 18, 22, 23, 25, 33, 37, 47, 49, 52, 53]
Study questions touching on several fields [11, 19]	No access to individual-level data [12, 16, 27]
Large sample size and greater statistical power [9, 23]	Large data granularity [21, 41]
No extra costs and time [11, 23]	Unobserved confounding [17, 18, 20–23, 33, 34, 38, 39, 46]
Explore complex relations [17]	Data of different AST methods is combined [9, 15]
Possible to assess impact of nationwide interventions [27]	Cautiousness needed with external validity [5]
Adjustment for other factors possible [35]	Completeness of data [4, 35]
	Not possible to link all entities [34, 40]

Some authors mentioned that a strength of data linkage studies is that they are very useful for hypothesis generating and that they can be used as a pilot study before undertaking more resource intensive prospective research. In addition, analysis can be improved when more data become available. Merging data also gives the opportunity to study questions touching on several fields, for example regarding the impact of sociologic and economic factors on health-related factors. Furthermore, it was stated that more output can be achieved from national surveillance data without extra costs and time, and associations between AMR determinants can be monitored in a longitudinal manner.

Thirteen papers mentioned the problem of ecological fallacy (Table 3). Ecological fallacy occurs when characteristics of a group are attributed to an individual [55]. Authors of eleven articles mentioned unobserved confounding as a limitation, which means that unmeasured variables affect both the independent variable and the outcome. Data on topics such as AMS and IPC protocols is scarce, so those subjects are often not taken into account [20]. Other limitations were that completeness of data reporting is a problem when using routinely collected data, and sometimes data cannot be included because not all entities could be linked. Also, AST can be performed in different ways and there is variation in antimicrobial susceptibility between the countries, but still these data is combined.

## Discussion

This systematic scoping review gives an overview of studies that linked AMR data and focuses on the yield and added value. Forty-eight AMR data linkage studies were identified. It was shown that data linkage studies allow researchers to integrate information from multiple sources to gain a more comprehensive understanding of AMR dynamics. Overall, the findings demonstrate that almost all studies (43/48) had added value and provided recommendations for clinical practice and future policies, or suggestions for further research or surveillance.

Identified AMR data linkage studies were divided into different categories based on the data sources that were used. The largest category consisted of studies linking AMR data to population characteristics. Population characteristics were also often linked to data on antibiotic use. Another large group linked AMR data to data on antibiotic use. To a lesser extent, AMR and/or antibiotic use data were linked to animal data, data on institutional characteristics or information on AMS. Types of data that were used most were AST data, prescription data, antimicrobial sales data and data from demographic databases. Fifteen studies linked data from different countries, and most of them linked data on a country level. Single-country studies mostly linked data on the regional level, but also on the level of healthcare facility or on individual patient level.

This review shows that there are only thirteen studies linking data on the level of the individual patient, indicating a notable gap in current research. The majority of included studies focused on linking data at a geographical level. Several factors can contribute to this predominance. For example, aggregated surveillance data are more often publicly available, standardized, and interoperable, while patient level data may be more restricted due to privacy concerns and often require additional approvals for use. Still, data linkage on patient level could be of added value, giving better insights into AMR dynamics than data linkage on an aggregated level. Therefore, there is a need for efforts directed towards better individual data access and management and overcoming legal hurdles complicating this type of research.

The added values of linkage studies differ across the identified categories. Studies involving patient characteristics more often lead to recommendations for clinical practice and implications for guiding future policies, while other categories more often lead to suggestions for further research. Recommendations for clinical practice often involve taking into account certain patient characteristics in AMS programs and antibiotic prescribing. Implications addressed to policy makers also frequently involve considering population characteristics and local and cultural context in campaigns, guideline development, and in targeting policy attention. Another added value of data linkage studies is that they can help in identifying risk factors associated with the development and spread of AMR as well as in assessing the effectiveness of interventions, since data on these factors is mostly captured in multiple databases. In addition, data linkage studies have proven to be very suitable for hypothesis generating. Because most data is already existing, data linkage studies can serve as preliminary investigation for further prospective research. Suggestions for further research are mostly already very specific. However, especially implications for policies and surveillance were often described as hints or suggestions.

AMR data linkage studies come with various limitations. For studies using AST data, combining data from different testing methods poses a challenge. Validation procedures are crucial to ensure data comparability. Another limitation is that studies had large data granularity and had no access to data at individual-level. Therefore, the majority of identified studies had ecological study designs, which are known for their difficulty in establishing causality [55, 56]. Moreover, ecological studies are very prone to ecological fallacy and unobserved confounding, which might affect the reliability and generalizability of findings, including recommendations for interventions targeting AMR. Despite these challenges, recommendations resulting from ecological data linkage studies can still be valuable and reliable if certain considerations are taken into account. First, researchers and policymakers should interpret findings with caution, recognizing the inherent limitations [56]. Ideally, recommendations from ecological studies should be supplemented with evidence from other study designs such as randomized controlled trials or cohort studies [57]. Also, researchers should conduct sensitivity analyses to assess the robustness of findings to potential biases [58]. Limitations that were identified in other medical data linkage studies not related to AMR are gaining and maintaining public trust for the use of data, reducing costs, and inefficiencies in how linked data are made available for research [59–61]. By considering and acknowledging the limitations, recommendations from both ecological and individual-level studies can still contribute to efforts to address AMR and improve public health outcomes.

Data linkage also has potential for public health related topics other than AMR. A recently published review described the use of linked data for infectious disease events and showed the variety of purposes the method can be used for [60]. The authors stated that data linkage is particularly useful for rare diseases affecting specific populations. Additionally, the World Health Organization published a report describing approaches to data linkage for evidence informed policy. It was mentioned that data linkage was used a lot during the COVID-19 pandemic and that the pandemic catalysed the secondary use of data [61]. Therefore, it would be interesting to look further into the lessons learned from COVID-19 data linkage research and apply them to AMR data linkage research. In both publications [60, 61], details on the method used for linkage, which was not covered in our review, were discussed. We initially aimed to gather more information on data linkage methods but found that the included articles offered limited details on this aspect. For AMR surveillance systems and other relevant data sources, more research should be performed to identify the practical methods for data linkage, for example ICT and governance possibilities.

This systematic scoping review has strengths and limitations. One of the strengths is the broad scope, which allowed us to include literature with different study designs, methodologies, and study outcomes. Therefore, this review provides an exhaustive overview of AMR data linkage studies. In addition, the process of a scoping review made it possible to use an inductive approach proceeding towards the added values of data linkage that were identified. However, several limitations also need to be acknowledged. First, the variability in terminology for data linkage, the lack of Medical Subject Headings and the absence of a general definition might have resulted in missing some relevant articles. However, we formulated a definition of data linkage to search for literature as uniform as possible. A second limitation is the absence of a formal risk of bias assessment. Scoping reviews map existing literature rather than assess outcomes, making a formal risk of bias assessment unnecessary. Moreover, traditional tools are often unsuitable for the diverse study designs and outcomes included. Lastly, some literature was older than five years, with the oldest study being published in 1998. Recommendations of newer studies might be more reliable due to better quality of surveillance data, advancements in ICT infrastructure, and incorporation of novel insights in methodologies. However, to answer our research question, we looked into the yield of the method of data linkage and were mostly interested in whether there is a yield and in what form.

## Conclusion

This systematic scoping review shows that data linkage on the subject of AMR is increasingly performed in recent years. Data linkage studies mainly lead to new hypotheses for future research and contribute to the optimisation of surveillance systems and interpretation of data in the context of guideline/policy development. There are, however, some limitations regarding ecological designs and data accessibility that need to be acknowledged and taken into account in practical deliveries. This systematic scoping review implicates that data linkage in the field of AMR has potential to gain a more comprehensive understanding of AMR dynamics. Therefore, more studies using data linkage, considering lessons learnt from COVID-19 data linkage studies, should be performed to improve knowledge on methodological approaches, data access, data management, and governance issues.

## Supplementary Information

Additional file 1.

Additional file 2.

## Data Availability

No datasets were generated or analysed during the current study.

## Abbreviations

AMR: Antimicrobial resistance
AMS: Antimicrobial stewardship
AST: Antimicrobial susceptibility testing
COVID-19: Coronavirus disease 2019
JIACRA: Joint inter-agency antimicrobial consumption and resistance analysis
NG-MAST: Neisseria gonorrhoeae multi-antigen sequence typing
PRISMA: Preferred reporting items for systematic reviews and meta-analysis
PRISMA-ScR: Preferred reporting items for systematic reviews and meta-analysis extension for scoping reviews
SGSS: Second generation surveillance system
UTI: Urinary tract infection
