# Supplementary material for: Enhancing antimicrobial resistance surveillance and research: a systematic scoping review on the possibilities, yield and methods of data linkage studies
Source: Antimicrob Resist Infect Control. 2025 Mar 29;14:25. doi: 10.1186/s13756-025-01540-7 (PMC11954275; doi:10.1186/s13756-025-01540-7)
Supplement: Supplementary file 2 — Additional file 2. [file 13756_2025_1540_MOESM2_ESM.docx]

**Additional file 2 - Search strategy**

**Embase.com**

| **No.** | **Query** | **Results** |
| --- | --- | --- |
| #15 | #6 AND #14 | 352 |
| #14 | #9 OR #13 | 276928 |
| #13 | #10 OR #11 OR #12 | 38510 |
| #12 | (('antimicrob*' OR 'antibioti*') NEAR/2 ('prescript*' OR 'prescrib*' OR 'treatment*' OR 'use' OR 'usag*')):ti | 25489 |
| #11 | (('antimicrob*' OR 'antibioti*') NEAR/2 ('optimalis*' OR 'stewardship*' OR 'policy' OR 'policies')):ti | 6209 |
| #10 | 'antimicrobial stewardship'/exp | 13633 |
| #9 | #7 OR #8 | 249589 |
| #8 | (('antibiotic' OR 'antimicrob*') NEAR/2 ('resistan*' OR 'drug resistan*' OR 'nonsusceptib*' OR 'non-susceptib*')):ti | 39316 |
| #7 | 'antibiotic resistance'/exp | 246209 |
| #6 | #1 OR #2 OR #3 OR #4 OR #5 | 33191 |
| #5 | ('data' NEAR/2 ('linked' OR 'linkag*' OR 'linking')):ti,ab | 22421 |
| #4 | ('ecologic*' NEXT/2 ('data' OR 'study' OR 'analys*')):ti | 3075 |
| #3 | 'panel data analys*':ti | 238 |
| #2 | ('data' NEAR/2 ('surveillance*' OR 'compil*' OR 'combin*' OR 'aggregat*')):ti | 3819 |
| #1 | ('data' NEAR/2 ('linked' OR 'linkag*' OR 'linking' OR 'integrat*' OR 'merged' OR 'merging' OR 'blended' OR 'blending' OR 'fusion' OR 'fused')):ti | 6318 |

**Scopus**

N = 192

( TITLE ( ( "data" W/2 ( "integrat*" OR "merged" OR "merging" OR "blended" OR "blending" OR "fusion" OR "fused" OR "surveillance" OR "compil*" OR "combin*" OR "aggregat*" ) ) OR "panel data analys*" OR ( "ecologic*" W/2 ( "analys*" OR "data" OR "study" ) ) ) OR TITLE-ABS ( "data" W/2 ( "linked" OR "linking" OR "linkag*" ) ) ) AND TITLE ( ( "antibiotic*" OR "antimicrob*" ) W/2 ( "resistan*" OR "nonsusceptib*" OR "non-susceptib*" OR "stewardship*" OR "optimilis*" OR "policy" OR "policies" OR "prescript*" OR "prescrib*" OR "treament*" OR "use" OR "usag*" ) )

**PubMed**

| **No.** | **Query** | **Results** |
| --- | --- | --- |
| #12 | #4 AND #11 | 129 |
| #11 | #7 OR #10 | 233305 |
| #10 | #8 OR #9 | 35599 |
| #9 | ("antibiotic*"[ti] OR "antimicrob*"[ti]) AND ("optimalis*"[ti] OR "stewardship*"[ti] OR "policy"[ti] OR "policies"[ti] OR "prescript*"[ti] OR "prescrib*"[ti] OR "treatment*"[ti] OR "use"[ti] OR "usag*"[ti]) | 34352 |
| #8 | "Antimicrobial Stewardship"[Mesh] | 4039 |
| #7 | #5 OR #6 | 205032 |
| #6 | ("antibiotic*"[ti] OR "antimicrob*"[ti]) AND ("resistan*"[ti] OR "nonsusceptib*"[ti] OR "non-susceptib*"[ti]) | 44327 |
| #5 | "Drug Resistance, Microbial"[Mesh] | 191696 |
| #4 | #1 OR #2 OR #3 | 14407 |
| #3 | "ecologic*"[ti] AND ("data"[ti] OR "analys*"[ti] OR "study"[ti]) | 6488 |
| #2 | "linked data"[tiab] OR "data linkag*"[tiab] OR "linking data"[tiab] | 6693 |
| #1 | "data"[ti] AND ("linked"[ti] OR "linkag*"[ti] OR "linking"[ti] OR "integrat*"[ti] OR "merged"[ti] OR "merging"[ti] OR "blended"[ti] OR "blending"[ti] OR "fusion"[ti] OR "fused"[ti] OR "surveillanc*"[ti] OR "compil*"[ti] OR "combin*"ti OR "aggregat*"[ti]) OR "panel data analys*"[ti] | 1250 |
